# The potential pathway from maternal intimate partner violence to early childhood development through height-for-age z-score: evidence from 10 low- and middle-income countries

**DOI:** 10.3389/fpubh.2026.1710561

**Published:** 2026-05-14

**Authors:** Lei Yue, Chunyu Li, Xinhong Zhu, Fen Yang, Guiyuan Qiao, Fei Huang

**Affiliations:** 1 School of Nursing, Hubei University of Chinese Medicine, Wuhan, China; 2 Hubei Shizhen Laboratory, Wuhan, China; 3 The First Clinical Medical School, Hubei University of Chinese Medicine, Wuhan, China; 4 Affiliated Hospital of Hubei University of Chinese Medicine, Wuhan, China; 5 Rehabilitation Medicine Center/Tuina Department, Hubei Provincial Hospital of Traditional Chinese Medicine, Wuhan, China

**Keywords:** child growth, domestic violence, early childhood development, height-for-age z-score, intimate partner violence

## Abstract

**Background:**

Intimate partner violence (IPV) experienced by mothers is negatively associated with early childhood development (ECD). However, whether IPV is related to ECD through height-for-age z-score (HAZ) remains unclear.

**Methods:**

This study combined cross-sectional data from the latest Demographic and Health Surveys conducted in 10 low-and middle-income countries, encompassing 4,448 mother–child dyads, with mothers aged 15 to 49 and children aged 36 to 59 months. ECD was evaluated using the early childhood development index. IPV was assessed using the modified Conflict Tactics Scale, including three forms of emotional, physical, and sexual IPV. And HAZ was calculated to reflect child growth. Mediation analyses were conducted to examine the pathways from IPV and its different forms to ECD through HAZ in total mother–child dyads, mother-girl dyads, and mother-boy dyads separately.

**Results:**

Any IPV was related to lower HAZ, which was further associated with higher likelihood of off-track development in total mother–child dyads and mother-girl dyads. Physical and sexual IPV were associated with ECD through HAZ in total mother–child dyads, mother-girl dyads, and mother-boy dyads. The indirect effects accounted for 7.2–9.2, 9.5–14.1, and 8.5–13.4% of the total effects of any, physical, and sexual IPV on ECD, respectively.

**Conclusion:**

The study suggested a potential pathway from IPV to ECD through HAZ. The findings highlight the importance of developing gender-tailored interventions targeting IPV to promote child growth and ECD.

## 1 Introduction

Early childhood development (ECD) includes four domains: socioemotional development, learning/cognition, literacy-numeracy, and physical development. Children who are developmentally on track in at least three of the four domains will be considered developmentally normal (1). Preschool children in low- and middle-income countries (LMICs) frequently do not reach their full developmental potential (2). A study showed that one-third of preschool children in LMICs failed to meet the basic milestones for cognitive or socioemotional development, while an additional 16% face setbacks in their physical development (3). Consequently, it is imperative to identify the influencing factors of ECD, so as to formulate targeted intervention measures.

Intimate partner violence (IPV) is defined as any form of violence within intimate relationships that may take the form of emotional, physical, or sexual violence (4). A substantial body of literature documented the negative impact of maternal IPV experiences on children’s development (5–8). Specifically, maternal experiences of IPV affected children’s physiological stress response systems, such as hypothalamic–pituitary–adrenal axis and autonomic nervous system, which was detrimental to the development of children’s fine and gross motor skills (9, 10). It was also linked to reduced executive function and poorer academic performance (11, 12). Moreover, children exposed to IPV had poorer emotion regulation, which in turn impaired their prosocial skills and hindered peer relationships and overall social functioning (13). Although the relationship between IPV and ECD was well established, the underlying mechanism remains unclear.

According to the conceptual framework proposed by Yount et al. (14), maternal experiences of IPV can hinder child growth through direct and indirect pathways. On one hand, children’s recurrent exposure to overt family conflict directly disrupts stress-responsive biological regulatory systems (15), thereby impairing nutrient absorption and compromising overall growth. On the other hand, IPV often constitutes a chronic and severe contextual stressor that compromises maternal caregiving capacity (16, 17)—often reflected in inadequate feeding practices and insufficient child healthcare practices—ultimately contributing to children’s nutritional deficiency and growth retardation. Height-for-age z-score (HAZ) is regarded as the best child growth indicator because it reflects cumulative linear growth (18). Consistent with the conceptual framework, the recent Demographic and Health survey (DHS) studies showed that children of mothers exposed to different forms of IPV were at a higher risk of impaired growth indicated by HAZ with less than −2 standard deviations of the median (15, 19–21).

In addition, a framework linking nutrition to mental development demonstrates that good nutritional status and growth can support the structure and functional activity of brain regions responsible for cognitive, motor, and socioemotional development, while enhancing interaction with the environment, thereby promoting these developmental outcomes (22). In line with the notion, substantial empirical studies found that impaired child growth, indicated by stunting or lower HAZ, can increase the risk of children’s cognitive, psychological language, and motor performance to deviate from the normal track (23–25), and the adverse impact of low HAZ on ECD exhibited long-term consequences (26).

Based on the existing theoretical frameworks, IPV may be related to ECD through HAZ. However, to the best of our knowledge, few empirical studies have investigated the pathway from IPV to ECD through HAZ. Therefore, this study aimed to use data from a representative sample of mother–child dyads in 10 LMICs to explore whether IPV first related to HAZ and further to ECD.

## 2 Methods

### 2.1 Participants

**Figure 1:**
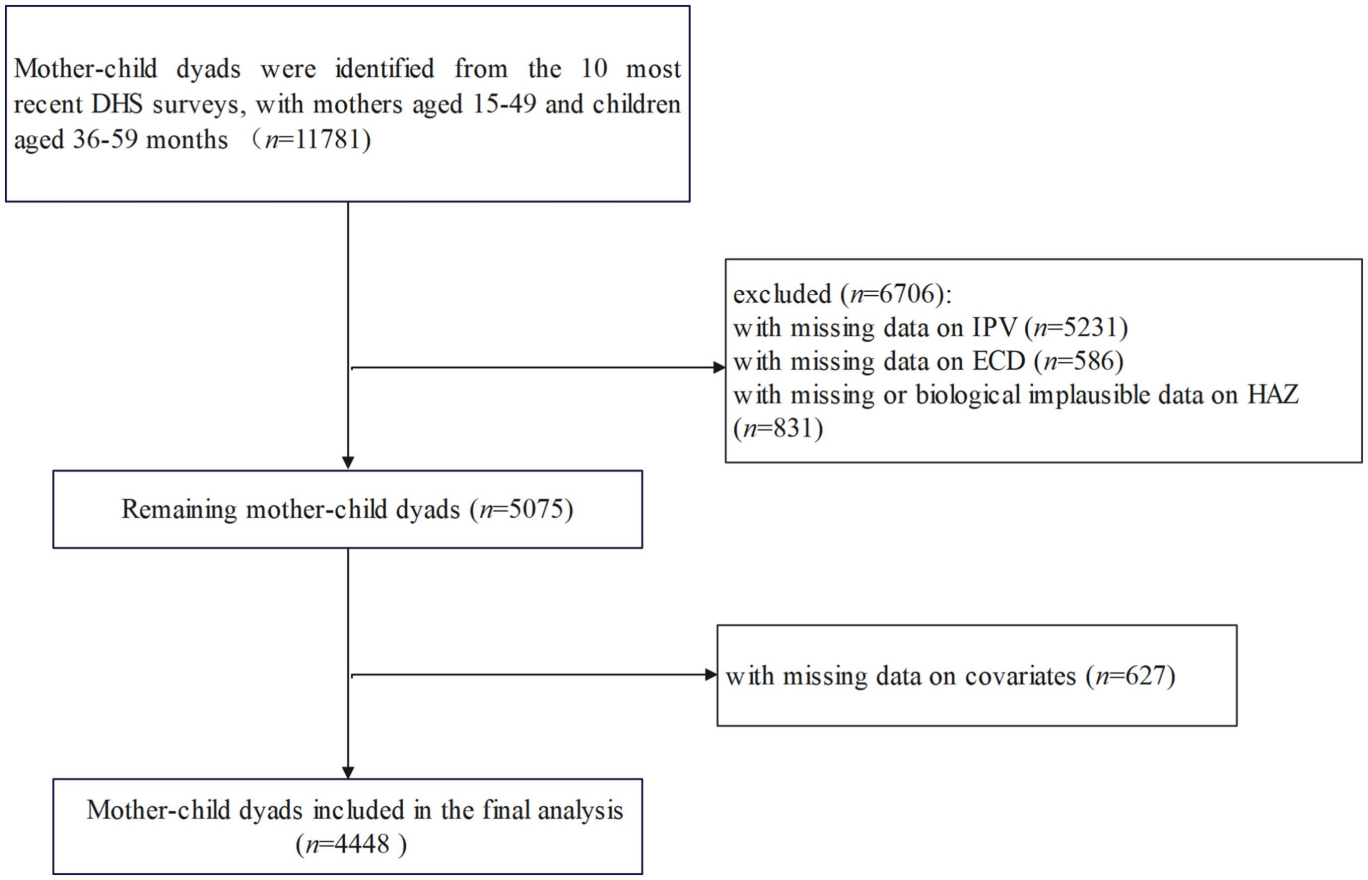
Flowchart of the sample selection. DHS, Demographic and Health Surveys; ECD, early childhood development; IPV, intimate partner violence; HAZ, height-for-age *z*-score.

The study used data from the latest publicly available DHS (27). The DHS is a nationally representative household survey that employs a multi-stage, stratified probability sampling method to collect health indicators of women of reproductive age (15 to 49 years), their children (0 to 59 months), and their households in LMICs (28). From the first year of the introduction of Early Childhood Development Index into the DHS (2011) to the time of analysis, only 10 DHS surveys included both the IPV questionnaire in women’s surveys and the Early Childhood Development Index questionnaire in children’s surveys, with consistent questionnaire items. Therefore, this study pooled the datasets from these 10 DHS countries into a cross-sectional dataset, comprising a sample of 11,781 children aged 36 to 59 months and their mothers aged 15 to 49 years. After excluding missing data for key variables and covariates, as well as implausible data for HAZ (greater than 6 or less than or equal to −6), 4,448 mother–child dyads were included. A complete list of the countries included in the analysis is provided in Supplementary Table S1. The sample-selection procedure is shown in Figure 1.

### 2.2 Measures

#### 2.2.1 Dependent variable

ECD was measured by the Early Childhood Development Index. It is a composite index, first introduced in united nations international children’s emergency fund fourth Multiple Indicator Cluster Survey in 2010. The Early Childhood Development Index comprises 10 yes/no questions, tailored for children of 36–59 months, aiming to evaluate four developmental domains: literacy-numeracy, physical development, learning/cognition, and socioemotional development (29). The literacy-numeracy domain (e.g., being able to identify at least 10 numbers) and the socioemotional development domain (e.g., getting along well with other children or adults) both contain 3 items. If at least two items are answer with “yes” in each domain, the child is deemed on track in that domain. A child is regarded as on track for physical development if they can pick up small object with two fingers and are not sometimes too sick to play. Likewise, a child is on track for learning/cognition if they can follow simple directions and perform task independently. And if a child is on track in at least three of the four domains, it indicates that ECD is on track.

#### 2.2.2 Exposure variable

The DHS domestic violence module is based on a modified Conflict Tactics Scale (CTS) and was used to assess IPV experienced by married or cohabitating women in the past 12 months on a dichotomized scale (0 = no, 1 = yes) (30). First, any IPV was defined as a positive response (i.e., yes) to at least one of the 13 items included in the module. The scale can be further classified into 3 subscales, namely, emotional IPV (3 items), physical IPV (7 items), and sexual IPV (3 items). If the answer of any item of a subscale is “yes,” it means that the mother experiences that form of IPV. Any IPV, emotional IPV, physical IPV, and sexual IPV were used as exposure variables separately.

#### 2.2.3 Mediator

HAZ is calculated by taking the difference between a child’s height and the median height for children of the same age and sex, then dividing that difference by the standard deviation of height for children of the same age and sex in the World Health Organization (WHO) reference population (31). A higher HAZ indicates better child growth. In accordance with the guidelines of WHO (18), HAZ greater than 6 or less than or equal to −6 was excluded.

#### 2.2.4 Covariates

Based on a review of literature (1, 3, 14, 19, 32–36), we identified several variables related to IPV, HAZ, and ECD as covariates, including maternal age, maternal highest level of education, maternal employment status, maternal current marital status, paternal age, paternal highest level of education, child’s age, child’s gender, child’s birth weight, child’s duration of breastfeeding, child’s birth order, household residence, household wealth, number of children under 5 years of age in the household, and country.

### 2.3 Statistical analysis

All statistical analyses were conducted using R (Version 4.4.1). The significance level was set at *α* = 0.05. In every DHS survey, the cluster codes and cluster sampling weights were provided in the datasets, so all analyses were performed on a weighted basis by “survey” package.

The weighted sample included 4,445 mother–child dyads. Sample characteristics were described using $\bar{x}\pm s$ for continuous variables, and frequency and percentage for categorical variables. The included and excluded samples were compared using *t*-tests or Chi-square tests. Then the *t*-tests were used to examine the differences in HAZ between children whose mothers experienced IPV and those whose mothers did not, as well as between children whose ECD were on track and those who were not. Chi-square tests were used to examine the bivariate relationship of IPV with ECD.

Then, mediation analyses were conducted using “mediation” package to examine whether any IPV was associated with ECD through HAZ after adjusting for covariates in total mother–child dyads. Considering the attributes of variables, the mediator was modeled using linear regression, and the dependent variable was modeled using logistic regression. Standard errors were estimated using cluster-robust variance estimation, and 1,000 Bootstrap iterations were performed to examine the significance of the indirect effect. Secondary analyses were conducted using emotional IPV, physical IPV, and sexual IPV as exposure variables separately to explore whether different forms of IPV exhibited distinct or similar associations with HAZ and ECD. In addition, considering that there were gender differences in the association between IPV and ECD (37), mediation analyses were repeated in mother-girl dyads and mother-boy dyads separately.

Finally, multiple group structural equation model was employed for sensitivity analysis to examine whether the pathway remained stable across different countries, with a chi-square difference test conducted to compare the unconstrained model (in which path coefficients were freely estimated) and the constrained model (in which path coefficients were constrained to be equal across all countries). A significant chi-square difference value indicates the presence of cross-country variations in the results.

## 3 Results

### 3.1 Sample characteristics

The characteristics of weighted samples are presented in Table 1. Over one-third of mothers (36.9%) experienced IPV in the past 12 months, with emotional IPV being the most prevalent (27.3%), followed by physical IPV (23.2%) and sexual IPV (8.1%). The mean age of mothers was 32.13 ± 6.80 years. Approximately 24.2% of the mothers had no formal education, and 44.4% had completed primary education, while nearly 70% were currently employed. Fathers showed slightly lower rates of no education (20.2%) and slightly higher rates of completed primary education (46.4%). The average age of fathers was 38.09 ± 9.51 years. Nearly half of children were male, and the mean age was 46.08 ± 6.76 months. Nearly two-thirds of children were not developmentally on track according to the Early Childhood Development Index. And the mean HAZ was −1.31 ± 1.28. 69.1% of the families resided in rural areas, and 46.5% were classified as poor based on the wealth index. Except for maternal current marital status, paternal age, residence, and number of children under 5 years of age, the remaining characteristics of included and excluded participants showed no significant differences (Supplementary Table S2).

**Table 1:** Sample characteristics.

Characteristics	*n* (%)	$\bar{x}\pm s$
Maternal		
Any IPV		
No	2,807 (63.1)	
Yes	1,638 (36.9)	
Emotional IPV		
No	3,230 (72.7)	
Yes	1,215 (27.3)	
Physical IPV		
No	3,414 (76.8)	
Yes	1,031 (23.2)	
Sexual IPV		
No	4,085 (91.9)	
Yes	360 (8.1)	
Age (year)		32.13 ± 6.80
Highest level of education		
No education	1,075 (24.2)	
Primary	1973 (44.4)	
Secondary	1,214 (27.3)	
Higher	183 (4.1)	
Employment status		
Not employed	1,420 (31.9)	
Employed	3,025 (68.1)	
Current marital status		
Married	2,911 (65.5)	
Living with partner	1,533 (34.5)	
Never in union/widowed/divorced/separated	0 (0.0)	
Paternal		
Age (year)		38.09 ± 9.51
Highest level of education		
No education	898 (20.2)	
Primary	2063 (46.4)	
Secondary	1,218 (27.4)	
Higher	266 (6.0)	
Child		
Gender		
Male	2,282 (51.3)	
Female	2,163 (48.7)	
Birth weight (kg)		3.20 ± 0.66
Duration of breastfeeding		
Never breastfed	107 (2.4)	
Ever breastfed	4,143 (93.2)	
Still breastfeeding	195 (4.4)	
Birth order		
First	855 (19.2)	
Second	1,009 (22.7)	
Third	848 (19.1)	
Fourth and beyond	1733 (39.0)	
Age (month)		46.08 ± 6.76
Height-for-Age Z-score		−1.31 ± 1.28
Development on track		
Not on track	2,603 (58.6)	
On track	1842 (41.4)	
Household		
Residence		
Urban	1,375 (30.9)	
Rural	3,070 (69.1)	
Wealth index		
Poor	2066 (46.5)	
Ordinary	890 (20.0)	
Rich	1,489 (33.5)	
Number of children under 5 years of age		
1	3,467 (78.0)	
2	773 (17.4)	
≥ 3	205 (4.6)	

IPV, Intimate partner violence. The final weighted sample is 4,445.

### 3.2 Bivariate analysis results

Compared with the children of mothers who had not experienced IPV, the children of mothers who had experienced IPV were more likely to be not developmentally on track, no matter what forms of IPV (any IPV: *χ^2^ =* 40.465, *p* < 0.001; emotional IPV: *χ^2^ =* 18.228, *p* < 0.001; physical IPV: *χ^2^ =* 33.509, *p* < 0.001; sexual IPV: *χ^2^ =* 26.566, *p* < 0.001). Children of mothers who experienced any, physical or sexual IPV were found to have lower HAZ than those whose mothers did not encounter these three forms of IPV (any IPV: *t =* 4.850, *p* < 0.001; physical IPV: *t =* 6.265, *p* < 0.001; sexual IPV: *t =* 5.129, *p* < 0.001). However, the bivariate relationship of emotional IPV with HAZ was not statistically significant (emotional IPV: *t =* 1.895, *p* = 0.058). And children whose ECD was on track had a higher HAZ than those with off-track ECD (*t =* −13.510, *p* < 0.001). See Table 2.

**Table 2:** Bivariate association of IPV with ECD and HAZ.

	Off-track ECD	On-track ECD	*t/χ^2^*	*p*	HAZ		
	*n* (%)/ $\bar{x}\pm s$	*n* (%)/ $\bar{x}\pm s$			$\bar{x}\pm s$	*t*	*p*
Any IPV			40.465	< 0.001		4.850	< 0.001
No (*n* = 2,807)	1,543 (55.0)	1,264 (45.0)			−1.24 ± 1.28		
Yes (*n* = 638)	1,060 (64.7)	578 (35.3)			−1.43 ± 1.27		
Emotional IPV			18.228	< 0.001		1.895	0.058
No (*n* = 3,230)	1829 (56.6)	1,401 (43.4)			−1.29 ± 1.31		
Yes (*n* = 1,215)	774 (63.7)	441 (36.3)			−1.37 ± 1.21		
Physical IPV			33.509	< 0.001		6.265	< 0.001
No (*n* = 3,414)	1919 (56.2)	1,495 (43.8)			−1.24 ± 1.26		
Yes (*n* = 1,031)	684 (66.3)	347 (33.7)			−1.53 ± 1.33		
Sexual IPV			26.566	< 0.001		5.129	< 0.001
No (*n* = 4,085)	2,346 (57.4)	1739 (42.6)			−1.28 ± 1.29		
Yes (*n* = 360)	257 (71.4)	103 (28.6)			−1.62 ± 1.19		
HAZ	−1.52 ± 1.31	−1.01 ± 1.18	−13.510	< 0.001			

IPV, Intimate partner violence; ECD, Early Childhood Development; HAZ, Height-for-Age Z-score.

### 3.3 Mediation analysis results

#### 3.3.1 The pathway from any IPV to ECD through HAZ

**Figure 2:**
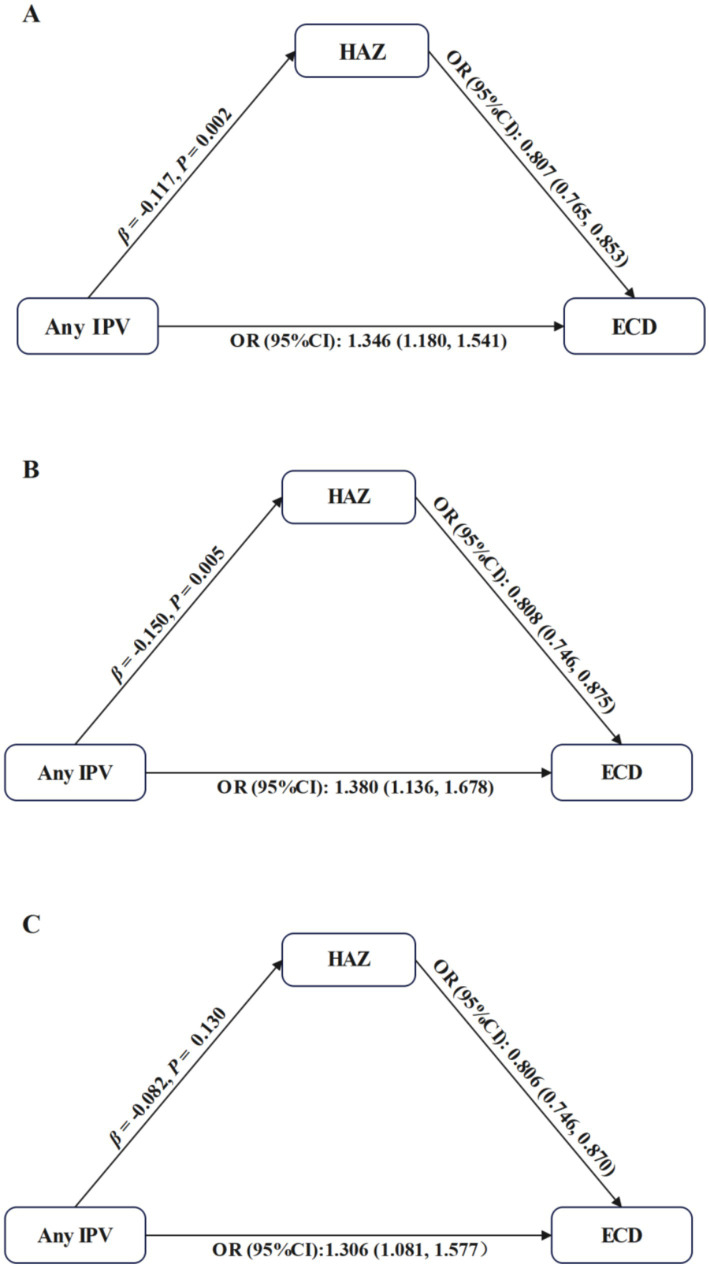
Mediation analysis for the pathway from any IPV to ECD through HAZ. IPV, Intimate Partner Violence; ECD, Early Childhood Development; HAZ, Height-for-Age *Z*-score. Each model was adjusted for maternal age, highest level of maternal education, maternal employment status, maternal current marital status, paternal age, paternal highest level of education, child’s age, child’s gender, child’s birth weight, child’s duration of breastfeeding, child’s birth order, household residence, household wealth, number of children under 5 years of age, and country. **(A–C)** represent results from total mother–child dyads, mother-girl dyads, and mother-boy dyads, respectively.

Any IPV was associated with lower HAZ (total mother–child dyads: *β* = −0.117, *p* = 0.002; mother-girl dyads: *β* = −0.150, *p* = 0.005), which was further related to higher likelihood of off-track development (total mother–child dyads: OR = 0.807, 95% CI: 0.765–0.853; mother-girl dyads: OR = 0.808, 95% CI: 0.746–0.875). The indirect effects accounted for 7.2% of the total effect of any IPV on ECD in total mother–child dyads and 9.2% in mother-girl dyads. However, the pathway from any IPV to ECD through HAZ was not statistically significant in mother-boy dyads (*β* = −0.082, *p* = 0.130; OR = 0.806, 95% CI: 0.746–0.870). See Figure 2 and Table 3.

**Table 3:** Indirect, direct, and total effects of IPV on ECD.

	Indirect effect		Direct effect		Total effect		Proportion mediated (%)
	Effect	*p*	Effect	*p*	Effect	*p*	
Total mother–child dyads							
Any IPV	0.005	0.006	0.064	< 0.001	0.069	< 0.001	7.2
Emotional IPV	0.002	0.350	0.050	0.002	0.052	< 0.001	3.8
Physical IPV	0.008	< 0.001	0.061	< 0.001	0.069	< 0.001	11.6
Sexual IPV	0.011	< 0.001	0.081	< 0.001	0.092	< 0.001	12.0
Mother-girl dyads							
Any IPV	0.007	0.004	0.069	0.002	0.076	< 0.001	9.2
Emotional IPV	0.003	0.312	0.048	0.032	0.051	0.032	5.9
Physical IPV	0.007	0.004	0.067	0.004	0.074	< 0.001	9.5
Sexual IPV	0.011	0.006	0.071	0.024	0.082	0.028	13.4
Mother-boy dyads							
Any IPV	0.004	0.120	0.057	0.018	0.061	< 0.001	6.6
Emotional IPV	0.001	0.752	0.051	0.026	0.052	0.026	1.9
Physical IPV	0.009	0.002	0.055	0.028	0.064	0.008	14.1
Sexual IPV	0.009	0.026	0.097	0.008	0.106	0.004	8.5

IPV, Intimate partner violence; ECD, Early Childhood Development.

#### 3.3.2 Secondary analysis results

##### 3.3.2.1 The pathway from emotional IPV to ECD through HAZ

**Figure 3:**
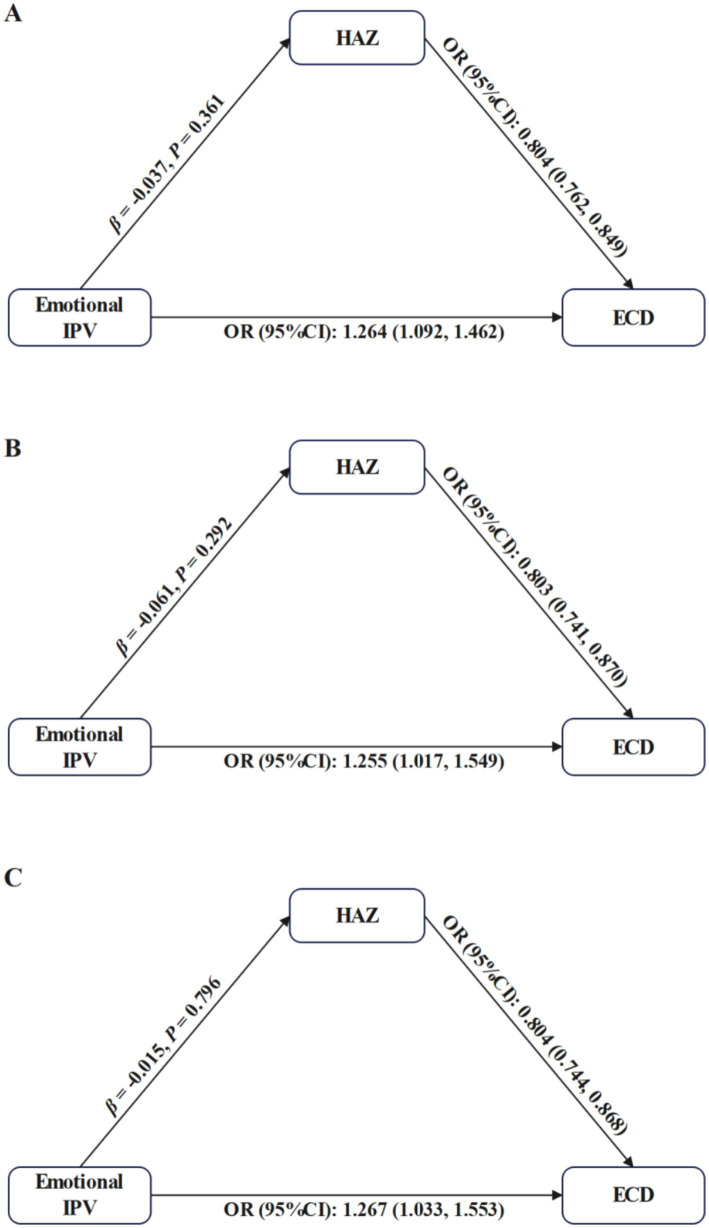
Mediation analysis for the pathway from emotional IPV to ECD through HAZ. IPV, Intimate Partner Violence; ECD, Early Childhood Development; HAZ, Height-for-Age Z-score. Each model was adjusted for maternal age, highest level of maternal education, maternal employment status, maternal current marital status, paternal age, paternal highest level of education, child’s age, child’s gender, child’s birth weight, child’s duration of breastfeeding, child’s birth order, household residence, household wealth, number of children under 5 years of age, and country. **(A–C)** represent results from total mother–child dyads, mother-girl dyads, and mother-boy dyads, respectively.

The pathway from emotional IPV to ECD through HAZ was not statistically significant in all dyads (total mother–child dyads: *β* = −0.037, *p* = 0.350; OR = 0.804, 95%CI: 0.762–0.849; mother-girl dyads: *β* = −0.061, *p* = 0.292; OR = 0.803, 95% CI: 0.741–0.870; mother-boy dyads: *β* = −0.015, *p* = 0.796; OR = 0.804, 95CI%: 0.744–0.868). See Figure 3 and Table 3.

##### 3.3.2.2 The pathway from physical IPV to ECD through HAZ

**Figure 4:**
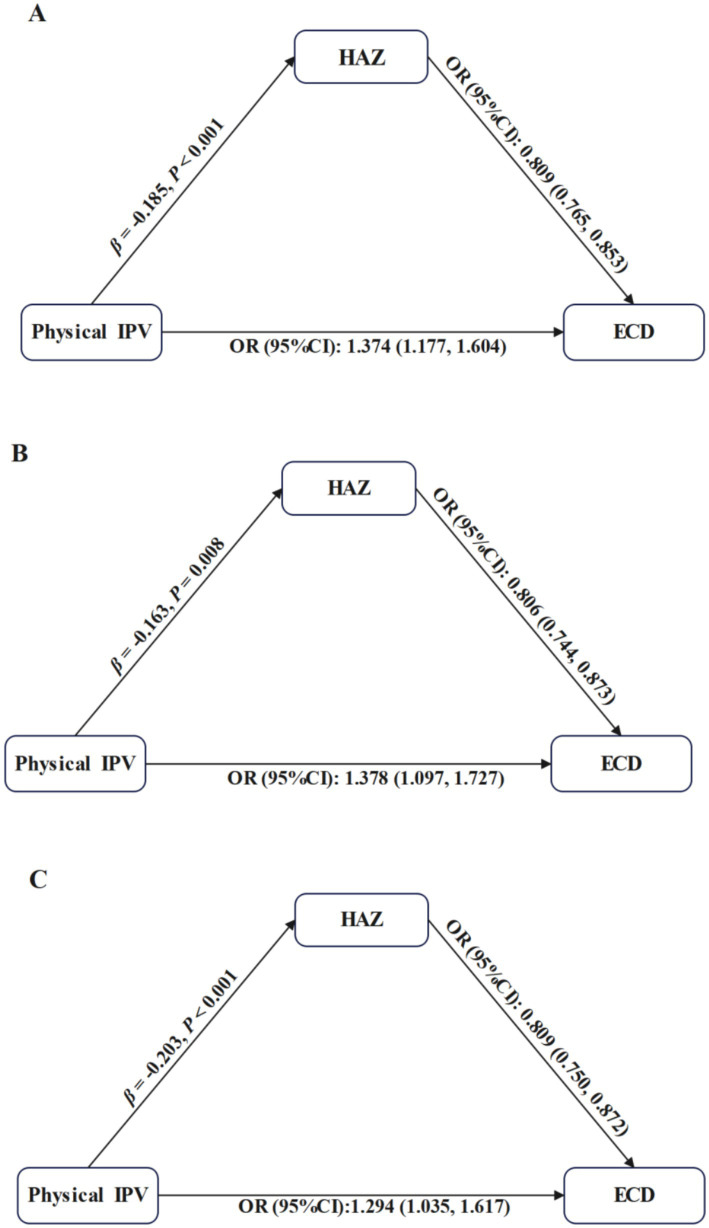
Mediation analysis for the pathway from physical IPV to ECD through HAZ. IPV, Intimate Partner Violence; ECD, Early Childhood Development; HAZ, Height-for-Age *Z*-score. Each model was adjusted for maternal age, highest level of maternal education, maternal employment status, maternal current marital status, paternal age, paternal highest level of education, child’s age, child’s gender, child’s birth weight, child’s duration of breastfeeding, child’s birth order, household residence, household wealth, number of children under 5 years of age, and country. **(A–C)** represent results from total mother–child dyads, mother-girl dyads, and mother-boy dyads, respectively.

Physical IPV was associated with lower HAZ (total mother–child dyads: *β* = −0.185, *p* < 0.001; mother-girl dyads: *β* = −0.163, *p* = 0.008; mother-boy dyads: *β* = −0.203, *p* = 0.001), and in turn, higher likelihood of being developmentally off-track (total mother–child dyads: OR = 0.809, 95% CI: 0.765–0.853; mother-girl dyads: OR = 0.806, 95% CI: 0.744–0.873; mother-boy dyads: OR = 0.809, 95% CI: 0.750–0.872). The indirect effects contributed to 11.6, 9.5, and 14.1% of the total effect of physical IPV on ECD in total mother–child dyads, mother-girl dyads and mother-boy dyads, respectively. See Figure 4 and Table 3.

##### 3.3.2.3 The pathway from sexual IPV to ECD through HAZ

**Figure 5:**
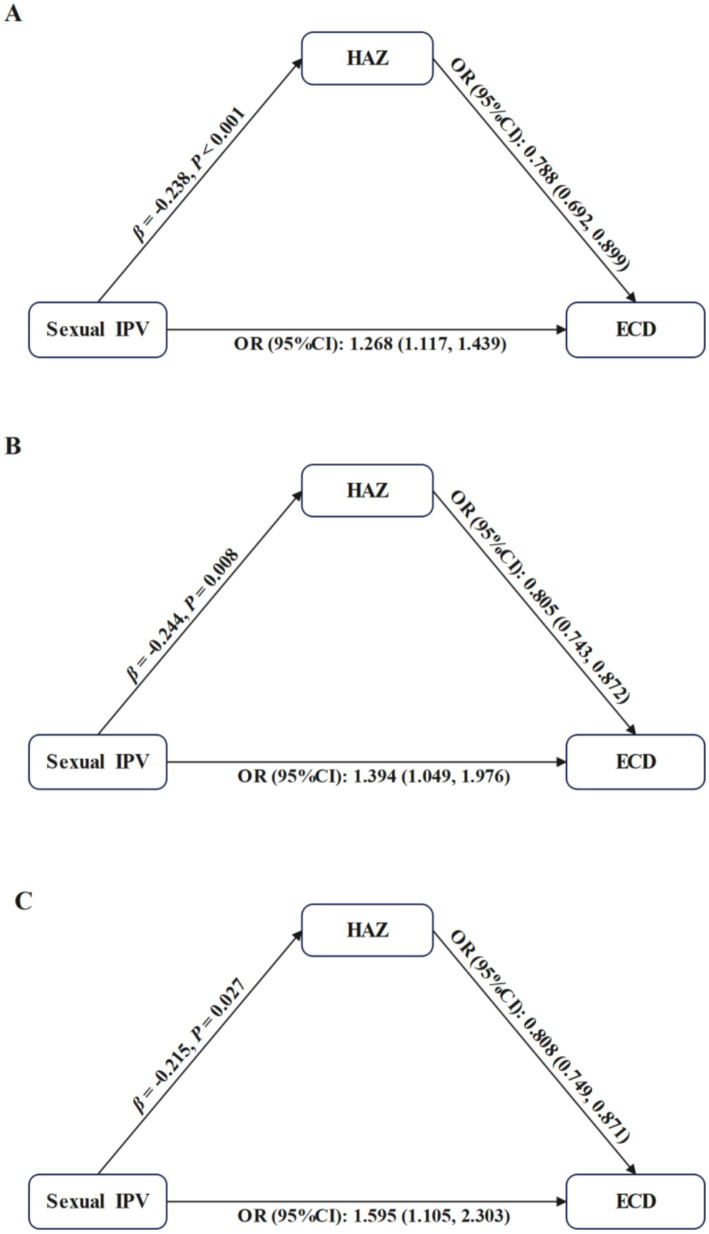
Mediation analysis for the pathway from sexual IPV to ECD through HAZ. IPV, Intimate Partner Violence; ECD, Early Childhood Development; HAZ, Height-for-Age Z-score. Each model was adjusted for maternal age, highest level of maternal education, maternal employment status, maternal current marital status, paternal age, paternal highest level of education, child’s age, child’s gender, child’s birth weight, child’s duration of breastfeeding, child’s birth order, household residence, household wealth, number of children under 5 years of age, and country. **(A–C)** represent results from tota–l mother–child dyads, mother-girl dyads, and mother-boy dyads, respectively.

Sexual IPV was related with lower HAZ (total mother–child dyads: *β* = −0.238, *p* < 0.001; mother-girl dyads: *β* = −0.244, *p* = 0.008; mother-boy dyads: *β* = −0.215, *p* = 0.027), which was associated with higher risk of off-track development (total mother–child dyads: OR = 0.788, 95% CI: 0.692–0.899; mother-girl dyads: OR = 0.805, 95% CI: 0.743–0.872; mother-boy dyads: OR = 0.808, 95% CI: 0.749–0.871). The indirect effects accounted for 12.0, 13.4 and 8.5% of the total effect of sexual IPV on ECD in total mother–child dyads, mother-girl dyads, and mother-boy dyads, respectively. See Figure 5 and Table 3.

### 3.4 Result of multiple group structural equation model

This study revealed no statistically significant cross-country differences in the pathways from any IPV (Δ*χ^2^ =* 8.814, *p =* 0.184), emotional IPV (Δ*χ^2^ =* 6.903, *p =* 0.330), physical IPV (Δ*χ^2^ =* 12.280, *p =* 0.056), and sexual IPV (Δ*χ^2^ =* 12.295, *p =* 0.056) to ECD through HAZ (Supplementary Table S3).

## 4 Discussion

The study found that any IPV was associated with lower HAZ and further with higher risk of off-track development in total mother–child dyads and mother-girl dyads. When considering different forms of IPV, only physical and sexual IPV were associated with ECD through HAZ in total mother–child dyads, mother-girl dyads, and mother-boy dyads. To the best of our knowledge, this is one of the first studies to examine the pathways from IPV and its different forms to ECD through HAZ, using data from mother–child dyads in 10 LMICs.

IPV was associated with lower HAZ, which is consistent with previous findings (38, 39). As primary caregivers, when mothers experience IPV, their children may also be exposed to violence (by witnessing or being informed about it), which can disrupt children’s biological stress response regulatory systems, leading to deficits or delays in physical growth (15, 40). In addition, IPV can reduce mothers’ stimulation and medical care for their children (6, 41, 42), which have been shown to exacerbate children’s stunting (43, 44). Finally, mothers experiencing IPV are more likely to suffer from mental health issues, such as depression and anxiety (45), leading to the neglect of children’s emotional needs and irregular feeding, thereby affecting the children’s nutrient absorption and hindering physical growth (46, 47).

In turn, children with lower HAZ were more likely to have off-track development. Adequate nutrition and normal growth provide the biological foundation for brain development, thereby promoting holistic cognitive, motor, and socioemotional development (22, 48). Inversely, lower HAZ can increase the likelihood of not being developmentally on track (26).

However, the pathway from emotional IPV to ECD through HAZ was not statistically significant. It may be because that compared to physical and sexual IPV, emotional IPV is more prevalent but generally less severe (49, 50). Severe IPV may chronically disrupt maternal metabolic and endocrine systems, thereby compromising breastfeeding quality or children’s nutrient absorption (51). It is worth noting that, the direct pathway from emotional IPV to ECD was still statistically significant, indicating that there might be others potential factor relating emotional IPV to ECD. For example, emotional IPV tends to correspond with poorer maternal mental health, which is tied to diminished quality in mother–child interactions, leading to long-term social, emotional, and cognitive challenges in children (52, 53). Future research is needed to further clarify the mechanisms underlying the relationship between emotional IPV and ECD.

Notably, the pathway from any IPV to ECD through HAZ was not statistically significant in mother-boy dyads, but was in mother-girl dyads. It is important to recognize that sex differences in linear growth patterns are well-documented (54, 55); for instance, boys generally exhibit lower HAZ than girls in early childhood, although this gap tends to narrow and may even reverse at later ages (54). These natural differences may partially lead to different outcomes between mother-boy dyads and mother-girl dyads in the IPV-HAZ-ECD pathway. In addition, previous research suggests that boys of mothers experiencing IPV exhibit lower hypothalamic–pituitary–adrenal axis activation and better self-regulation than girls of mothers experiencing IPV (56). We hypothesize that differential biological stress responses may partially contribute to the sex-specific finding, although this remains to be tested in future studies. Another possible explanation is that limited financial, material, and social resources in LMICs may lead parents to engage in more gender-based differential treatment of their children, including disparities in nutritional resource allocation (57–59). The relationship between gender norms and child nutrition and growth may be complex and vary across countries, and also requires further empirical exploration. Finally, we observed that mothers of girls reported a higher prevalence of experiencing multiple types of IPV compared to mothers of boys, which may partially explain the sex-specific pathway. Future research should examine whether the coexistence of different types of IPV has differential impacts on child growth by sex.

The findings indicate that the integration of IPV intervention into child growth and nutrition promotion may represent a potential strategy for preventing adverse ECD. The intergrated program has been preliminarily implemented in LMICs, such as father-engaged home-visiting program linked to social protection system (60). It is committed not only to providing psychoeducation on child development and nutrition promotion, but also to reducing family violence by father engagement and improved conflict resolution and parental emotion regulation skills (60). Empirical studies showed that the father-engaged home-visiting program can reduce IPV and enhance child’s nutritional status and ECD in Rwanda (60, 61). In addition, pediatric healthcare providers, as frontline practitioners, play a pivotal role in identifying children at risk of off-track development related to maternal IPV and low HAZ. For mothers screening positive, nutritional interventions should be delivered to address growth deficits, such as delivering tailored feeding guidance and responsive caregiving support for mothers. Meanwhile, pediatric healthcare providers can facilitate referrals of mothers exposed to IPV to specialized violence services through cross-sectoral collaboration.

There are several limitations of the study. First, the cross-sectional design is unable to establish causal links between IPV, HAZ, and ECD. Therefore, the significant IPV-HAZ-ECD pathway reflects statistical mediation rather than causal mediation. Second, the representativeness of the sample is limited, and thus the results cannot be generalized to all LMICs. Additionally, the data were self-reported, and hence might lead to report bias and recall bias, especially in communities with IPV stigma. Finally, the included covariates are limited to the available data collected in the DHS, and other unmeasured confounders might bias the observed pathway from IPV to ECD through HAZ. The E-value was calculated to assess the extent to which the pathway may be confounded by unmeasured confounders associated with mediator and outcome (62). We found that an unmeasured confounder associated with HAZ and ECD with minimum approximate odds ratio of 1.8-fold each, above the measured covariates, could explain the observed pathway, but weaker confounders could not.

## 5 Conclusion

This study contributes to the existing literature by revealing the role of HAZ in the association between IPV and its different forms and ECD among mother–child dyads from LMICs. More specifically, any IPV was associated with lower HAZ, which was further related to higher likelihood of not being developmentally on track in total mother–child dyads and mother-girl dyads. The pathways from physical and sexual IPV to ECD through HAZ existed in total mother–child dyads, mother-girl dyads, and mother-boy dyads. The findings highlight the importance of gender-tailored interventions targeting IPV to promote child growth and early development. In addition, given that HAZ is easy to obtain and cost-effective, it is suitable for resource-limited LMICs to inform early management of children whose early development is not on track.

## Data Availability

The original contributions presented in the study are included in the article/Supplementary material, further inquiries can be directed to the corresponding authors.
