# Supplementary material for: The potential pathway from maternal intimate partner violence to early childhood development through height-for-age z-score: evidence from 10 low- and middle-income countries
Source: Front Public Health. 2026 May 14;14:1710561. doi: 10.3389/fpubh.2026.1710561 (PMC13216183; doi:10.3389/fpubh.2026.1710561)
Supplement: Supplementary file 1 [file Supplementary_file.DOCX]

**Table S1**. Sample size and percentage distribution by country and survey year

| Country | Year | *n* (%) |
| --- | --- | --- |
| Benin | 2017-2018 | 466 (10.5) |
| Burundi | 2016-2017 | 297 (6.7) |
| Cameroon | 2011 | 281 (6.3) |
| Haiti | 2016-2017 | 526 (11.8) |
| Honduras | 2011-2012 | 1388 (31.2) |
| Maldives | 2016-2017 | 388 (8.7) |
| Rwanda | 2019-2020 | 307 (6.9) |
| Senegal | 2019 | 196 (4.4) |
| Timor-Leste | 2016 | 348 (7.8) |
| Togo | 2013-2014 | 248 (5.6) |

**Table S2**. Comparison of Characteristics between the included and excluded participants

| Characteristics | Included  *n (*%*)/*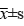 | Excluded  *n (*%*)/*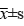 | *t/χ^2^* | *P* |
| --- | --- | --- | --- | --- |
| **Maternal** |  |  |  |  |
| Any IPV |  |  | 1.586 | **0.208** |
| No | 2809 (63.2) | 1952 (61.6) |  |  |
| Yes | 1639 (36.8) | 1218 (38.4) |  |  |
| Emotional IPV |  |  | 0.096 | **0.757** |
| No | 3232 (72.7) | 2285 (73.9) |  |  |
| Yes | 1216 (27.3) | 798 (26.1) |  |  |
| Physical IPV |  |  | 1.446 | **0.229** |
| No | 3416 (76.8) | 2412 (78.9) |  |  |
| Yes | 1032 (23.2) | 648 (21.1) |  |  |
| Sexual IPV |  |  | 1.426 | **0.232** |
| No | 4088 (91.9) | 2752 (92.1) |  |  |
| Yes | 360 (8.1) | 236 (7.9) |  |  |
| Age (year) | 32.21 ± 6.79 | 32.36 ± 6.93 | -0.782 | **0.434** |
| Highest level of education |  |  | 2.071 | **0.558** |
| No education | 1075 (24.2) | 1792 (24.5) |  |  |
| Primary | 1974 (44.4) | 3350 (45.8) |  |  |
| Secondary | 1215 (27.3) | 1821 (24.9) |  |  |
| Higher | 184 (4.1) | 351 (4.8) |  |  |
| Employment status |  |  | 6.117 | **0.054** |
| Not employed | 1420 (31.9) | 2499 (35.6) |  |  |
| Employed | 3028 (68.1) | 4521 (64.4) |  |  |
| Current marital status |  |  | 68.427 | < 0.001 |
| Married | 2918 (65.6) | 3335 (47.7) |  |  |
| Living with partner | 1530 (34.4) | 1594 (22.8) |  |  |
| Never in union/  widowed/  divorced/separated | 0 (0.0) | 2021 (29.5) |  |  |
| **Paternal** |  |  |  |  |
| Age (year) | 45.86 ± 6.72 | 45.38 ± 6.90 | 2.078 | 0.038 |
| Highest level of education |  |  | 3.576 | **0.311** |
| No education | 898 (20.2) | 1489 (20.9) |  |  |
| Primary | 2064 (46.4) | 3412 (47.9) |  |  |
| Secondary | 1220 (27.4) | 1653 (23.2) |  |  |
| Higher | 266 (6.0) | 620 (8.7) |  |  |
| **Child** |  |  |  |  |
| Gender |  |  | 0.049 | **0.825** |
| Male | 2284 (51.4) | 3752 (51.2) |  |  |
| Female | 2164 (48.6) | 3581 (48.8) |  |  |
| Birth weight (kg) | 3.21 ± 0.69 | 3.22 ± 0.71 | -0.792 | **0.429** |
| Duration of breastfeeding |  |  | 0.992 | **0.609** |
| Never breastfed | 107 (2.4) | 202 (2.8) |  |  |
| Ever breastfed | 4145 (93.2) | 6582 (90.1) |  |  |
| Still breastfeeding | 196 (4.4) | 518 (7.1) |  |  |
| Birth order |  |  | 2.266 | **0.519** |
| First | 856 (19.2) | 1482 (20.3) |  |  |
| Second | 1010 (22.7) | 1625 (22.2) |  |  |
| Third | 848 (19.1) | 1102 (15.1) |  |  |
| Fourth and beyond | 1734 (39.0) | 3124 (42.4) |  |  |
| Age (month) | 38.00 ± 9.19 | 37.79 ± 9.41 | 0.614 | **0.539** |
| Height-for-Age Z-score | -1.31 ± 1.27 | -1.30 ± 1.32 | 0.009 | **0.993** |
| Development on track |  |  | 0.362 | **0.548** |
| Not on track | 2604 (58.5) | 3825 (56.7) |  |  |
| On track | 1844 (41.5) | 2922 (43.3) |  |  |
| **Household** |  |  |  |  |
| Residence |  |  | 8.076 | 0.004 |
| Urban | 1378 (31.0) | 2852 (39.0) |  |  |
| Rural | 3070 (69.0) | 4481 (61.0) |  |  |
| Wealth index |  |  | 0.107 | **0.948** |
| Poor | 2066 (46.4) | 3482 (47.5) |  |  |
| Ordinary | 890 (20.0) | 1525 (20.8) |  |  |
| Rich | 1492 (33.6) | 2326 (31.7) |  |  |
| number of children under 5 years of age |  |  | 80.156 | < 0.001 |
| 1 | 3473 (78.1) | 3681 (70.2) |  |  |
| 2 | 774 (17.4) | 1056 (20.1) |  |  |
| ≥ 3 | 201 (4.5) | 506 (9.7) |  |  |

Note: IPV, Intimate partner violence. All analyses were based on unweighted sample sizes. *P*-values were presented in bold if there were no statistically significant differences in characteristics between included and excluded participants.

**Table S3**. Cross-country differences in pathways from IPV to ECD through HAZ

| Exposure variable | Model type | RMSEA | CFI | TLI | SRMR | Δ*χ^2^* (Δ*df*) | *P* |
| --- | --- | --- | --- | --- | --- | --- | --- |
| Any IPV | Unconstrained | 0.027 | 0.987 | 0.918 | 0.007 | 8.814 (6) | 0.184 |
|  | Constrained | 0.025 | 0.984 | 0.926 | 0.008 |  |  |
| Emotional IPV | Unconstrained | 0.018 | 0.994 | 0.962 | 0.006 | 6.903 (6) | 0.330 |
|  | Constrained | 0.017 | 0.993 | 0.967 | 0.007 |  |  |
| Physical IPV | Unconstrained | 0.021 | 0.992 | 0.948 | 0.006 | 12.280 (6) | 0.056 |
|  | Constrained | 0.024 | 0.986 | 0.935 | 0.007 |  |  |
| Sexual IPV | Unconstrained | 0.006 | 0.999 | 0.996 | 0.005 | 12.295 (6) | 0.056 |
|  | Constrained | 0.016 | 0.994 | 0.971 | 0.007 |  |  |

Note: IPV, Intimate partner violence; ECD, Early Childhood Development; HAZ, Height-for-Age Z-score; RMESA, Root Mean Square Error of Approximation; CFI, Comparative Fit Index; TLI, Tucker-Lewis Index; SRMR, Standardized Root Mean Square Residual.
